# Chinese parents’ school-readiness beliefs and parenting styles: patterns and associated factors

**DOI:** 10.3389/fpsyg.2023.1279175

**Published:** 2024-01-09

**Authors:** Shujing Cui, David Greger

**Affiliations:** Institute for Research and Development of Education, Faculty of Education, Charles University, Prague, Czechia

**Keywords:** school readiness, parenting style, parents’ school-readiness beliefs, latent profile analysis, socioeconomic status (SES), COVID-19 study

## Abstract

**Introduction:**

A smooth transition to primary school is positively related to children’s later school experience. Certain parental school-readiness beliefs and parenting styles, among other factors, contribute to the smoothness of this transition.

**Methods:**

Therefore, this study adopted a latent profile analysis to examine the patterns of Chinese parents’ school-readiness beliefs and their parenting styles and investigated socioeconomic status (SES) differentials in both. Two-stage probability sampling method is adopted in this study and a total of 1,204 Chinese parents of 5- to 6 years-old children were investigated with school-readiness beliefs scale, Parenting Styles and Dimensions Questionnaire, as well as scale of attitudes regarding roles in school readiness All data analyses were processed in Mplus 8.6.

**Results and discussion:**

Three profiles were identified: (1) supportive parenting with a very strong emphasis on school readiness; (2) partially supportive parenting with a reflection of school readiness; (3) weakly supportive parenting with no emphasis on school readiness. Higher SES was found to be more likely to be associated with membership in Profile 1 rather than Profile 2. The present study shows quantitative support for Anette Lareau’s work and has implications for the development of more targeted parental intervention programs.

## Introduction

The successful transition from pre-primary school to primary school lays a solid foundation to children’s later school success (Ghaye and Pascal, 1989; Burrell and Bubb, 2000). A multitude of studies stress the contribution of family contexts to children’s school readiness both theoretically and empirically. From the perspective of bio-ecological theory (Bronfenbrenner, 2005), family contexts are placed in the micro-system surrounding the transition process and exert the most proximal effect on a child’s transition to school. Parents’ school-readiness beliefs, what parents perceive as important competence of their child for school readiness, and parental involvement in getting their child ready for school both constitute important factors in such contexts. The academic socialization model (Taylor et al., 2004) postulates that parents’ beliefs on school readiness have an impact on children’s transition outcomes through parental involvement such as transition practices. Empirical studies show supportive evidence for such effects. On one hand, children’s transition outcomes like school-readiness skills and academic achievement growth are found to be positively linked to their parents’ school readiness beliefs (Barbarin et al., 2008; Puccioni, 2015): children performed better in school-readiness domains that their parents valued highly (Barbarin et al., 2008). Additionally, parental involvement contributes to successful transition to school and well-being of children (Puccioni, 2018; Heel et al., 2020; Ng et al., 2020; Slicker et al., 2021; Polat and Bayındır, 2022; Cui, 2023). Further exploration of the underlying mechanism of this effect showed that parental school-readiness beliefs could influence a child’s transition to school by mediating parental involvement, and they thus provide an important indirect impact on children’s school readiness (Sy and Schulenberg, 2005; Puccioni, 2015). Consequently, parents’ school-readiness beliefs are a contributor to children’s school readiness. As a feature of parenting practices, parenting style also directly influences children’s development. Research on Chinese kindergarten parents shows associations between parenting style and children’s school-readiness outcomes (Xia et al., 2020; Xia, 2022). The study of Xia et al. (2020) reveals a negative association between the authoritarian parenting style and children’s socioemotional school-readiness outcomes as well as a positive association between authoritative parenting and school readiness, including both academic and socioemotional outcomes. Qualitative evidence shows that certain combinations of parenting styles and school-readiness beliefs could be associated with socioeconomic status (SES) groups (Lareau, 2002), though quantitative evidence is lacking. Thus, it is necessary to identify subgroups of parents by taking into consideration both factors with a quantitative and person-centered approach. As parents’ school-readiness beliefs and parenting style are associated with children’s school-readiness outcomes and may be more amenable to change compared to other factors like SES, which is associated with children’s academic achievement, exploring this topic could be critical for parental intervention programs aiming to tackle equity in the transition to primary school.

Given the above, the present study takes a person-centered approach to explore typologies based on the combination of Chinese parents’ school-readiness beliefs and their parenting style, as well as factors associated with profile memberships. We conducted a latent profile analysis based on a large sample of parents of children in the last year of kindergarten, before entering primary school, in Chongqing, China. Given that Chinese parents’ school-readiness beliefs are understudied, the present study aims to add to the existing literature by delineating their school-readiness beliefs and parenting styles. This could also help reveal the complexity of factors associated with child school readiness and how they associate in subgroups of parents. Last but not least, identifying subgroups of parents holding certain school-readiness beliefs and displaying certain parenting styles would facilitate the recognition of subgroups of families in which children are at a higher risk of being unready, conducive to more effective parental intervention programs.

### Parents’ school-readiness beliefs

A body of research has documented the variability of parents’ school-readiness beliefs (Piotrkowski et al., 2000; Sy and Schulenberg, 2005; Hatcher et al., 2012; Puccioni, 2015) in various contexts and across cultures. Parents in different cultures hold divergent school-readiness beliefs. Basically, the main feature distinguishing different school readiness beliefs lies in the tensions between academic skills and social-emotional learning. On one hand, such divergence could be related to the cultural tradition, for instance, Asian tradition stressing the importance of academic learning more than European. As it is revealed in the study by Sy and Schulenberg (2005), Asian American parents espouse stronger school-readiness beliefs in the value of academic or cognitive skills compared to European American parents. Australian migrant parents with Asian background value academic and social development most and focus less on emotional learning and communication skills (Binasis et al., 2022). On the other hand, the preschool system feature could also play a role in the divergence of school-readiness belief across cultures. In some preschool systems, play-oriented pedagogy is the tradition, while in others, academic-focus tendency has been a long history. In more play-oriented preschool system, parents value the social-emotional learning more than academic learning. For instance, in Danmark, the tradition of preschool focuses on play as well as the social learning of children. Danish parents deem social learning to be the most important skills for a child’s school readiness, while academic competence is not important as it could grow naturally (Kjaer et al., 2020). While, in many Asian preschool systems including China, academic-focus pedagogical practices are more culturally rooted than play-oriented paradigm (Bautista et al., 2021), findings on parents’ school readiness beliefs show different features. A few studies show that Chinese parents generally value children’s academic skills and personal qualities or approaches to learning, like self-confidence and perseverance, interest in learning, etc. (Zhang et al., 2008; Chan, 2012). These studies delineate the characteristics of school-readiness beliefs of Chinese parents. However, their findings were based on relatively small and nonrepresentative samples of about 200 parents and covered two regions of China (Hong Kong and the City of Changchun); given the cultural and geographic diversity of China and its large population, the current study tries to add to existing literature and better understand Chinese parents’ school-readiness beliefs by drawing on a representative sample from the City of Chongqing, China.

Most studies examine parents’ readiness beliefs using a variable-centered approach (Abry et al., 2015; Puccioni, 2015), measuring the variable as homogeneous and continuous, while some research reveals that parents’ school-readiness beliefs could be discontinuous in nature. There could be different subgroups of parents holding different patterns of school-readiness beliefs even in a given sociocultural context. Barbarin et al. (2008) collected qualitative data from parents in a disadvantaged community in the US and showed that several patterns emerged in terms of parents’ responses. For instance, some parents emphasize a single domain while others stress multiple domains. The most common combination pattern was highlighting general knowledge, social competence or self-regulation, whereas one rare pattern placed a very high expectation on academic skills. Besides, some quantitative research reveals that among US immigrant parents from China, the Dominican Republic and El Salvador, two profiles of parents’ school-readiness beliefs were found; the first emphasizes academic skills and the second emphasizes learning-related skills (Sawyer et al., 2022). Kim et al. (2005) identifies three subgroups of parents with a representative sample in the US by using cluster analysis, namely, *typical, high standards* and *low academic emphasis* groups. Whereas, a latent profile analysis in another study with a representative sample in the US found that there are two main subgroups of parents who hold distinct school-readiness beliefs, namely. High expectation and very high expectation (Slicker et al., 2021). The exploratory nature of cluster analysis and latent profile analysis as well as parents’ beliefs may change over the decades, and sample characteristics could lead to inconsistent research findings. Such studies with a person-centered approach contributed to our knowledge of the heterogeneity of parents’ school-readiness beliefs and shed light on parental intervention programs, as certain school-readiness beliefs could be associated with parental involvement and thus with children’s school readiness. However, in extant research, to our knowledge, few studies have addressed Chinese parents’ school-readiness beliefs from a person-centered perspective, thus very little is known about the patterns of Chinese parents’ school-readiness beliefs and the characteristics associated with certain patterns.

Consistent with multiple dimensions of a child’s school-readiness competence, which usually includes cognitive and language development, social-emotional skills, behavioral adjustment, physical and health development, approaches to learning, etc. (Gai, 2007; Janus and Offord, 2007; Hughes et al., 2015), parents’ school-readiness beliefs are multi-dimensional as well. Among prior studies on the patterns or profiles of parents’ school-readiness beliefs, only a few probed school-readiness beliefs in terms of the multidimensional conceptualization of school readiness (Piotrkowski et al., 2000; Sawyer et al., 2022), whereas most studies focused on parents’ beliefs in a narrower scope of school readiness, mainly regarding academic and social-emotional skills (Slicker et al., 2021). Although Sawyer et al. (2022) consider more than 5 domains of parents’ school-readiness beliefs, their study only includes 63 US immigrant parents, of which 22 are Chinese with a relatively high SES; such sample characteristics limit the generalization of results.

### Parents’ school-readiness beliefs, parenting style, attitudes regarding roles in school readiness, and SES

Given the fundamental role school transition plays in a child’s later school experience, it is crucial to level the playing field for children at school entry to promote equality in education. However, disparities in children’s school readiness were identified across socioeconomic status (SES) spectrums in many countries including China (Fitzpatrick et al., 2014; Larson et al., 2015; Wolf and McCoy, 2019; Ren et al., 2020). Such gaps could be derived partly from the variation of parents’ school-readiness beliefs related to SES, which result in a gap between parental involvement and children’s school readiness. According to the academic socialization model, parents’ beliefs, transition practices and children’s transition outcomes are shaped by socioeconomic and cultural contexts (Taylor et al., 2004). Thus, SES could be associated with parents’ school-readiness beliefs. Though it is revealed that parents’ school-readiness beliefs vary within cultural and socioeconomic contexts, such as ethnicity (Barbarin et al., 2008; Puccioni, 2018) and the education level of parents (West et al., 1993), the association between patterns of such beliefs and SES had not been adequately studied, especially with a quantitative approach.

Besides, an association between parents’ school-readiness beliefs, parenting style and SES has been documented by qualitative studies. Parenting style is used to describe a typology of features in parenting practice. Researchers categorize parenting style into four types: authoritative, authoritarian, permissive and negligent parenting, according to two dimensions of parenting behavior: parental responsiveness and control (Baumrind, 1971; Maccoby and Martin, 1983). Authoritative parenting features high control and high responsiveness, authoritarian parenting is characterized as high control and low responsiveness. Permissive parenting is low in control and high in responsiveness, while negligent parenting is low in both control and responsiveness. Authoritative parenting is mainly characterized by democratic parenting, encouraging autonomy, and setting up clear standards for children’s conduct, while authoritarian parenting displays more directive parenting, forceful and punitive discipline, and an expectation of obedience to parental authority (Gafoor and Kurukkan, 2014; Asio and College, 2023). Authoritative and authoritarian parenting practices, linked with specific school-readiness beliefs, are potentially featured in subgroups of parents, as shown in qualitative studies. For instance, findings from well-known qualitative research highlight that parents from disadvantaged backgrounds or from minority ethnic groups had a higher propensity of using authoritarian and directive parenting strategies and emphasized more knowledge of facts and self-regulatory readiness skills (e.g., obedience), than their middle-class counterparts (Lareau, 2002, 2011; Tobin and Arzubiaga, 2013). Similarly, Barbarin et al. (2008) reported in their research that parents in a high-need community who hold traditional views of children and authoritarian views of control tend to have narrow views of school readiness and parents who are high in the use of directive strategies have the tendency to emphasize the importance of knowledge for school readiness (Barbarin et al., 2008). Thus, from a person-centered perspective, we could expect certain combinations of parenting style and parents’ school-readiness beliefs to be identified in the population of parents, and in turn, have an impact on children’s school-readiness outcomes. However, such an association is still to be tested with quantitative studies.

How parents perceive roles of their own and school settings in school readiness constitute another aspect of parents’ beliefs relevant to children’s school readiness. Peterson et al. (2018) investigated the attitudes regarding roles in school readiness among US latino parents with low income and found that they emphasize their own role in school readiness much more than that of schools. However, such attitudes are underexplored in extant literature, with very few studies focusing on the topic. And to the best of our knowledge, Chinese studies concerning such attitudes are lacking. However, it is worth examination as attitudes of roles in school readiness could be a proxy for the extent of parents’ willingness of investment in facilitating their children’s school readiness.

### Present study

Numerous studies have examined parents’ school-readiness beliefs or parenting style as factors associated with a child’s school readiness, using a variable-centered approach. However, smaller subgroups of parents with different school-readiness beliefs and parenting style could not be identified in variable-centered studies due to the major focus on the average level of variables of interest. Besides, as subgroups of parents with a specific combination of school-readiness beliefs and parenting style could potentially exist based on previous studies (Lareau, 2002, 2011; Barbarin et al., 2008; Tobin and Arzubiaga, 2013), it would be more informative to use a person-centered approach to delineate a more nuanced relationship of the two factors associated with children’s school readiness. Additionally, this study adds to the existing literature by investigating Chinese parents’ school-readiness beliefs using a person-centered approach on a large probability sample, which was seldom addressed previously. Consequently, this study is guided by the following questions and hypotheses:

1. How many groups of Chinese parents could be identified based on their school-readiness beliefs and parenting styles (authoritarian and authoritative)? What are these groups like?
2. Are SES and other family demographics and child characteristics associated with patterns of parents’ school-readiness beliefs and parenting style? If yes, how are they associated with profiles based on parents’ school-readiness beliefs and parenting style?
3. How do the identified subgroups of parents view their own roles and the school’s role in school readiness?

Generally speaking, this study is exploratory research, and its hypotheses are described in a broader manner as follows. For the first question, we expect to find 3 to 4 groups with a distinct combination of parents’ school-readiness beliefs and parenting style. However, as very few studies have explored this subject with a person-centered approach, the number of groups is an open question. Furthermore, we hypothesize that one group of parents in China might hold more academic-oriented school-readiness conceptions, endorse more self-regulatory skills, display more authoritarian and less authoritative parenting. And one group of parents could be less academic-oriented, stress social-emotional skills and approaches to learning, show more authoritative and less authoritarian parenting. For the second question, as prior studies showed, we hypothesize that the lower SES would be associated with high authoritarian, low authoritative parenting and with school-readiness conceptions that emphasize more academic-oriented and self-regulatory skills. In contrast, higher SES would be associated with high authoritative, low authoritarian parenting, with parents’ beliefs placing more emphasis on social–emotional readiness. For the third question, we do not formulate a specific hypothesis as this question is more exploratory than confirmatory.

## Materials and methods

### Research design

The present study adopted a cross-sectional design to address the above research questions. We surveyed parents’ school readiness beliefs, their parenting styles, SES, and their attitudes about roles in school readiness simultaneously, as these variables could be relatively stable, thus, we inspected them only once in our study. The data collection was conducted during COVID-19 pandemic, starting from November 2022 to March 2023, with intermittent interruptions caused by repeated closures of kindergartens.

### Participants

To generate representative samples, we drew samples from Chongqing in China, which is one of four municipalities directly under the central government of China (the other three are Beijing, Shanghai and Tianjin). The sampling frame is parents of kindergarteners before school entry (children aged 5 to 6 years old) in about 5,660 kindergartens in the city of Chongqing. The rationale is that parents of children in the last year of ECEC are much more likely to think about the transition to school than parents of younger children because the school entry time is approaching. It would be easier to elicit their valid school-readiness beliefs. We drew a two-stage probability sample.

We collected data mainly through online self-completion questionnaires. Before administering the questionnaire survey, we contacted the principals of selected kindergartens in person or by phone, to clarify the research purpose and ask for their consent to participate in the study. At the first stage, we randomly selected and contacted 45 kindergartens, of which 35 agreed with participation. At the second stage, all eligible parents were surveyed. Online self-completion questionnaires were sent to the principal of each kindergarten, who distributed the questionnaires to all parents of children in the last year of kindergarten before school entry. For parents who were not able to fill in the online questionnaire due to limited access to internet and mobile devices, pen-and-paper questionnaires were delivered.

Thus, the final sample consisted of 1,204 parents of kindergarteners in the last year before school entry in Chongqing. The response rate at the kindergarten level was 86%, and the response rate for parents’ questionnaires was roughly 63%. Detailed characteristics of the final sample of parents are presented in Table 1; the sample covers a wide range of SES levels.

**Table 1:** Demographic characteristics and socioeconomic status (*N* = 1,204).

Demographic characteristics and socioeconomic background	M (SD)/*N*(%)
Age of respondent	31.00 (5.88)
Gender	
Boy	616 (51%)
girl	588 (49%)
Age of child in months	71.76 (6.34)
Birth order	
Only child	416 (34%)
First born but not the only child	252 (21%)
Second born	507 (42%)
Third born	24 (2%)
other	5 (0.4%)
Parents’ highest level of education	
Primary education	8 (1.7%)
Lower secondary education	115 (15.3%)
Upper secondary education	259 (24.1%)
Post-secondary, non-tertiary education	275 (20.6%)
Bachelor’s or equivalent level	467 (33.2%)
Master’s degree	70 (4.4%)
Doctor’s degree	10 (0.6%)
Parents’ highest level of occupation	
Has never worked outside home for pay, general laborer, or semi-professional (skilled agricultural or fishery worker, craft or trade worker, plant or machine operator)	76 (6%)
Clerical (clerk or service or sales worker)	335 (27.8%)
Small business owner	194 (16.1%)
Professional (corporate manager or senior official, professional, or technician or associate professional)	599 (49.8%)
Number of books at home	
0–10	152 (12.6%)
11–25	254 (21.1%)
26–100	481 (40.0%)
101–200	165 (13.7%)
More than 200	152 (12.6%)
Number of children’s books at home	
0–10	156 (13.0%)
11–25	265 (22.0%)
26–100	291 (24.2%)
101–200	285 (23.7%)
More than 200	207 (17.2%)
Annual traveling occurrences before COVID-19	
Never	361 (30.0%)
Once	383 (31.8%)
Two to three times	371 (30.8%)
More than three times	89 (7.4%)
Annual household income	
Less than USD 776 (<5,000 RMB)	39 (3.2%)
USD 776–3,104 (5000–20,000 RMB)	135 (11.2%)
USD 3105–7,761 (20001–50,000 RMB)	135 (11.2%)
USD 7762–12,417 (50001–80,000 RMB)	135 (11.2%)
USD 12418–15,522 (80001–100,000 RMB)	194 (16.1%)
USD 15522–21,828 (100001–150,000 RMB)	177 (14.7%)
USD 21829–29,104 (150001–200,000 RMB)	159 (13.2%)
USD 29105–43,656(200001–300,000 RMB)	128 (10.6%)
More than USD 43656 (More than 300,000 RMB)	102 (8.5%)
Socioeconomic status (SES)	8.9 (2.33)

## Measures

### School-readiness beliefs

The current scale used for surveying parents’ school-readiness beliefs was adapted based on the item pool in the existing literature. The battery of items was mainly selected from scales and interview responses in 6 studies (Piotrkowski et al., 2000; Barbarin et al., 2008; Abry et al., 2015; Mullis and Martin, 2017; Puccioni, 2018; Sawyer et al., 2022). Scales in two of these studies were used to measure Chinese respondents’ school-readiness beliefs or used internationally, which lend insight into the development of the scale in our study (Mullis and Martin, 2017; Sawyer et al., 2022). In the aforementioned 6 scales, school-readiness beliefs were measured in several domains, including beliefs on the importance of children’s academic competence, social-emotional skills, self-regulatory behaviors, approaches to learning or interest/engagement, as well as self-care/independence, though some studies focused on only 2 to 3 dimensions. Based on the theoretical dimensions of school readiness and the above dimensions for measuring school-readiness beliefs in extant literature, the scale in the present study was structured with 5 domains, namely academic, social-emotional, self-regulatory, approaches to learning, and self-care.

The preliminary scale included 33 items covering 5 domains. Translation into Chinese and back-translation were used to adapt the English scale to the Chinese version. Then the scale in Chinese was read by experts in the Chinese language, kindergarten teachers and experts in education to ensure coherence and avoid ambiguity or the over-complication of statements. The scale requires the respondents to rate each item from 1 to 5, ranging from not important to very important, according to their own perception on the importance of specific skills for a child’s school readiness.

We conducted an exploratory factor analysis before the survey with the data of a pilot study, which included 240 parents of kindergarteners in the last year before school entry. However, in partial contrast to our theoretical construct, the exploratory factor analysis yielded a final 4-factor solution that encompassed 14 items. The four factors were academic competence (3 items, including count by oneself, knows characters, etc.), approaches to learning (3 items, including self-confidence, has patience, etc.), self-regulatory competence (4 items, such as pays attention, follows directions), and social-emotional competence (4 items, including takes turns, communicates needs verbally, etc.). A confirmatory factor analysis of the 4-factor measurement model showed indices that adequately fit the current sample (TLI = 0.96, CFI = 0.97, RMSEA = 0.083, SRMR = 0.03). Item loadings across the 4 dimensions range from 0.67 to 0.95, which are considered strong associations. Cronbach alphas for the 4 domains are all above 0.90, and Cronbach alpha for the whole scale is 0.95.

### Parenting style

Parenting style is measured by the Parenting Styles and Dimensions Questionnaire (PDSQ) (Robinson et al., 2001), which is a five-point Likert-type scale. In the present sample, we only used the authoritarian and authoritative subscale, and based on the confirmatory factor analysis of the sample, we also excluded one item from the original authoritarian subscale in the measurement model to achieve a good fit. The authoritative subscale covers the dimensions of connection (5 items), regulation (5 items) and autonomy granting (5 items) in parenting practices. The final authoritarian subscale used in the present study measures the dimensions of physical coercion (3 items), verbal hostility (4 items) and non-reasoning/punitive (4 items) in parenting practices.

The confirmatory factor analysis showed a good fit for the authoritarian subscale. Most of the fit indices fell into the range of a good fit (CFI = 0.933, TLI = 0.91, GFI = 0.92, SRMR = 0.04. RMSEA = 0.091 [0.077, 0.10]). Item loadings of the authoritarian scale range from 0.45 to 0.85, indicating moderate to strong associations. The authoritative subscale shows good validity in the current sample as well, with all fit indices falling into the range of a very good fit (CFI = 0.961, TLI = 0.953, GFI = 0.932, SRMR = 0.04, RMSEA = 0.057 [0.045, 0.069]). Item loadings of the authoritative scale range from 0.58 to 0.80, indicating strong associations. Cronbach alpha of the authoritarian subscale is 0.91, while that of the authoritative subscale is 0.87, indicating good reliability.

### Parents’ attitudes regarding their roles in school readiness

Parents’ attitudes regarding their roles in school readiness were measured with a scale of 5 items. The items of this scale were adapted from the subscale used in the 2007 School Readiness Parent Survey of the US Department of Education National Household Education Surveys Program (National Center for Education Statistics, 2007; Peterson et al., 2018). The relevant attitudes were measured using 5-point Likert-type items, asking about how parents rate their own and the school’s responsibilities for their child’s school readiness. The exploratory factor analysis yielded a two-factor solution. The two factors were termed family role and school role, respectively. The family role includes 3 items, requiring parents to rate the importance and their own responsibility for preparing their child for school, for instance: “Preparing my child for school is important to me and my family.” The school role covers 2 items, asking parents about their opinion on the responsibility of kindergartens and primary schools in preparing children for school, for instance: “Preparing my child for school is the responsibility of kindergarten.” A confirmatory factor analysis showed that factor loadings of the items ranged from 0.86 to 0.97, indicating high factor loadings. The confirmatory factor analysis factor model showed a good fit (CFI = 0.99, TLI = 0.98, GFI = 0.98, SRMR = 0.02, RMSEA = 0.061 [0, 0.17]). The reliability of the whole scale is acceptable, with Cronbach *α* = 0.77.

### Socioeconomic status

SES is measured with multiple items, including parents’ education, occupation, the possession of books at home, household income, as well as the annual frequency of family traveling before COVID-19. The items about parents’ education, occupation and books in household are well-developed indicators for SES used in TIMSS 2019 (Mullis and Martin, 2017). Family travel occurrences annually and household income are added as indicators for SES in the present study as well. Socioeconomic status (SES) is measured in this study by the highest level of occupation of parents, the highest level of education of parents, the number of children’s books in the home, the number of books in the home, and the household income. The composite score of the above items is computed via a principal component analysis.

### Family demographic characteristics

Demographic information regarding children and parents was also requested via the parents’ questionnaire, including the child’s gender, age, their birth order as well as the caregiver’s age.

### Analytic approach

Given that latent profile analysis is advantageous for addressing research questions concerning qualitatively configural differences that involve many variables, which are not easily realized by other techniques (Spurk et al., 2020), the present study mainly adopts this method for its data analysis. To answer the first research question, we used latent profile analysis to distinguish between groups of parents based on parents’ school-readiness beliefs and parenting style because this model-based statistics method allows us to identify underlying homogeneous subgroups in the population of parents and capture as much variation as possible between groups. Then, to answer research question 2, we tested the hypothesis about predictors (SES and demographic characteristics) for profile membership for different combinations of parental school-readiness beliefs and parenting style by applying the three-step approach (Vermunt, 2010; Asparouhov and Muthén, 2014) of latent profile analysis. The first step is to fit the model and identify the underlying latent classes. The second step is to assign individuals to classes based on posterior probabilities. In the final step, the covariates were used to predict latent profile membership, using the assigned profile as the indicator variable for the new latent class model. To answer the third research question, we used descriptive statistics with an inferential Wald equality test of means (Wang et al., 2005; Asparouhov, 2007) to compare differences between groups’ means. All analyses were processed in Mplus 8.6 (Muthén and Muthén, 2017).

## Results

### Latent profiles of parents’ school-readiness beliefs and parenting style

Latent profile analysis was conducted to identify latent profiles based on parents’ school-readiness beliefs and parenting style. Table 2 shows the comparisons of fit indices for 2- to 4-profile solutions. We mainly relied on the BIC for model comparison and choice of the appropriate model, given the good performance and consistency of this index for selecting the correct model with larger sample sizes (Tofighi and Enders, 2008). Besides, we also take into consideration the percentage of cases assigned to each profile and the conceptual interpretation and meaningful classification of profiles (Ram and Grimm, 2009). As Table 2 shows, the AIC, BIC, and aBIC are the lowest, with three-profile solutions in comparison with other solutions, indicating an optimal model fit, as smaller values indicate a better model fit regarding these indices (Geiser, 2013). Meanwhile, the entropy of the three-profile solution is 0.94, which shows accuracy in assigning parents to profiles and good separation between the three profiles (Geiser, 2013). Regarding the profile size, the additional profile in the three-profile solution contains more than 1% of the total sample size and more than 25 cases, which is acceptable (Lubke and Neale, 2006). Both the BLRT and the LMRLRT favor a three-profile solution over a two-profile solution (2*∆LL = 2538.89, *p* < 0.0001), and a four-profile solution would further improve on the three-profile solution. However, when taking into consideration the above indices and the conceptual interpretability of the solution, we decided that the three-profile solution is the optimal model.

**Table 2:** Fit indices for latent profile analysis based on parents’ school-readiness beliefs and parenting style.

Model and profile	Count	Proportion	Entropy	AIC	BIC	aBIC	LMRLRT (p)	BLRT (p)
Two-profile	879	0.73	0.89	12021.84	12123.71	12060.18	3633.76 (<0.0001)	3804.52 (<0.0001)
	325	0.27						
Three-profile			0.94	9498.96	9641.57	9552.63	2424.93 (<0.0001)	2538.89 (<0.0001)
	861	0.71						
	312	0.26						
	31	0.03						
Four-profile			0.95	9514.96	9698.32	9583.97	835.06 (<0.001)	874.30 (<0.0001)
	399	0.39						
	317	0.31						
	276	0.27						
	31	0.03						

AIC, Akaike information criteria; BIC, Bayesian information criteria; aBIC, sample size-adjusted BIC; BLRT, bootstrapped likelihood ratio test; LMR-LRT, Lo-Mendell-Rubin likelihood ratio test.

Table 3 shows the mean values of the school-readiness beliefs and parenting style for the three latent profiles. We named the three profiles based on the means of school-readiness beliefs and parenting style indicators to highlight the characteristics of each underlying subgroup of parents. We conducted Wald tests of parameter constraints to examine whether the pair-wise mean differences of the indicators across profiles are statistically significant. To control the risk of Type I error, we use the Bonferroni (1936) correction to interpret the results of Wald tests. We applied a Bonferroni (1936) correction for the interpretation of the results of Wald tests. We compared means for each profile indicator three times (1 v. 2, 1 v. 3, 2 v. 3). Thus, we use an alpha of (*α* = 0.017 (0.05/3 = 0.017) when determining significant differences of means shown in Table 3. As shown in Table 3, means of the indicators differ significantly across profiles except that of authoritarian parenting.

**Table 3:** Mean values for the three latent profiles.

Variables	Overall sample M (SE)	Profile1: Supportive parenting with a very strong emphasis on school readiness (71%) M (SE)	Profile2: Partially supportive parenting with a reflection of school readiness (26%) M (SE)	Profile3: Weakly supportive parenting with no emphasis on school readiness (3%) M (SE)
School-readiness beliefs				
Academic	4.27 (0.02)	4.56 (0.02)	3.76 (0.03)	1.30 (0.10)
Approaches to learning	4.52 (0.02)	4.78 (0.02)	4.15 (0.03)	1.13 (0.09)
Social emotional	4.58 (0.02)	4.91 (0.01)	4.05 (0.02)	1.03 (0.04)
Self-regulatory	4.60 (0.02)	4.89 (0.01)	4.17 (0.02)	1.09 (0.06)
Parenting style				
Authoritarian parenting	2.12 (0.02)	2.12 (0.02)^a^	2.10 (0.04)^a^	2.19 (0.12)^a^
Authoritative parenting	4.11 (0.02)	4.26 (0.02)	3.78 (0.03)	3.44 (0.10)

We applied a Bonferroni (1936) correction for the interpretation of the results of Wald tests. We compared means for each profile indicator three times (1 v. 2, 1 v. 3, 2 v. 3). Thus, we use an alpha of *α* = 0.017 (0.05/3 = 0.017) when determining significant differences of means. Profiles differed at *p* < 0.02 unless noted. Superscripts indicate that the differences were not significant at *p* < 0.02.

As displayed in Table 3, Profile 1 is characterized as *Supportive parenting with a very strong emphasis on school readiness* and constitutes 71% of parents. Profile 2 features *Partially supportive parenting with a reflection of school readiness* and is less prevalent in comparison with Profile 1, with 26% parents in the population belonging to this class. Profile 1 and Profile 2 feature a somewhat lower emphasis on the importance of concrete academic skills compared to other domains. The smallest proportion (3%) of parents belong to Profile 3, which is characterized as *Weakly supportive parenting with no emphasis on school readiness*. Notably, academic skills are rated slightly higher relative to the other domains in Profile 3.

**Figure 1:**
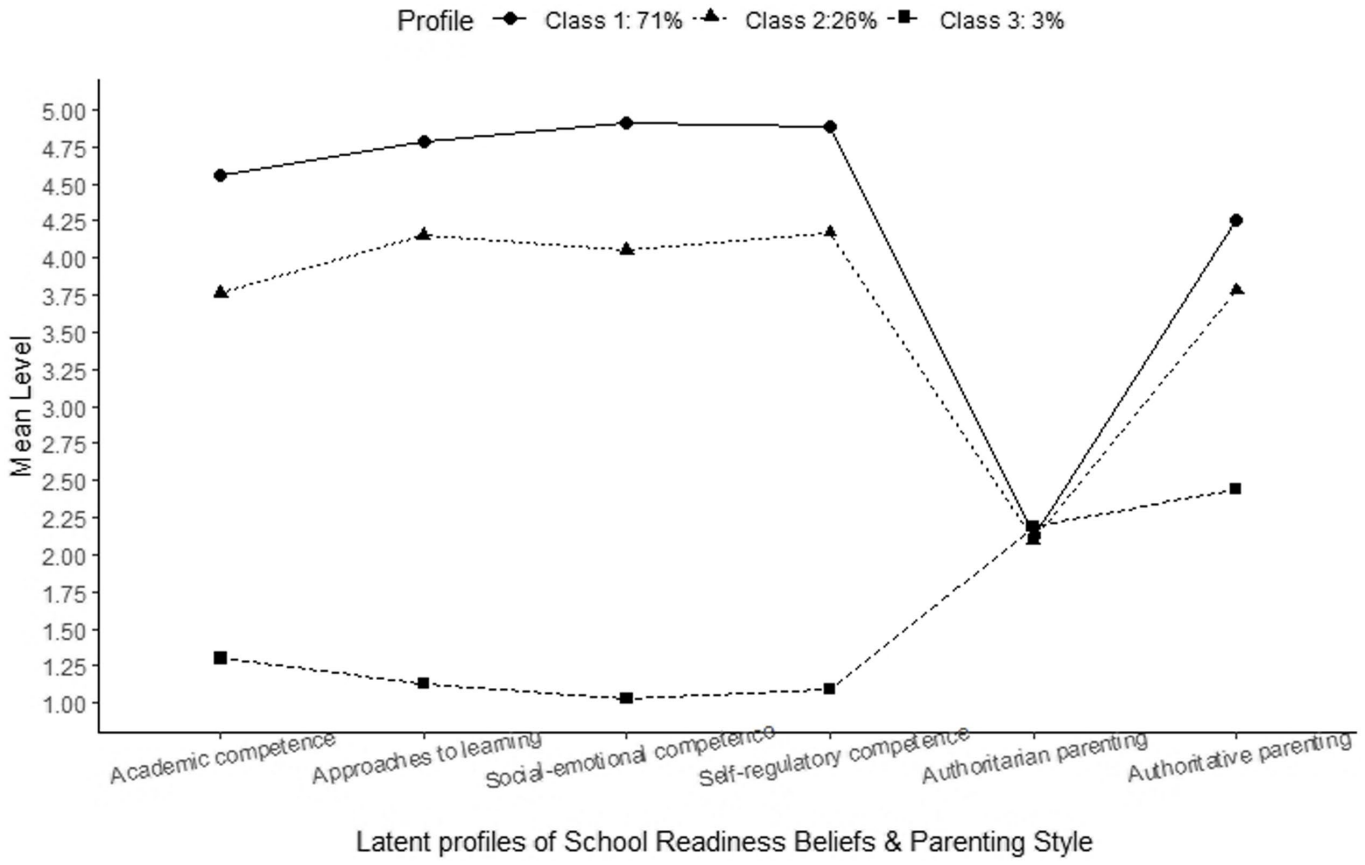
Latent profiles of parents’ school-readiness beliefs and parenting style.

For the overall sample, as Table 3 shows, the mean scores of school-readiness beliefs and parenting style show a high level of overall emphasis on the school-readiness competence of children, a low frequency of authoritarian parenting, and a high frequency of authoritative parenting. However, the three profiles evince obvious heterogeneity regarding school-readiness beliefs, and different levels of overall expectations across all four domains and different levels of authoritative parenting are displayed across the three groups. Despite the distinct features, the two most prevalent profiles display some common patterns. Profile 1 and Profile 2 both rated academic competence as the least important. Profile 1 ranked social-emotional competence as the most important, while Profile 2 deemed self-regulatory skills the most important. Profile 3 rated social-emotional competence as the least important. Both Profile 1 and Profile 2 rated the domains of self-regulatory and approaches to learning as important. Figure 1 shows the visual depiction of the latent profiles of parents’ school-readiness beliefs and parenting style. Common to all profiles is a low value of authoritarian parenting, while the level of authoritative parenting is the highest for Profile 1 and lowest for Profile 3. For Profile 1, parents strongly emphasize the importance of their children’s school-readiness skills with mean scores of over 4.5 across all domains and display a low value of authoritarian parenting contrasted with the highest score for authoritative parenting (mean score of 4.26), indicating they most frequently engage in democratic parenting, encouraging autonomy for their children. For Profile 2, parents still hold high expectations but place less emphasis on the importance of children’s school-readiness skills compared to Profile 1, with mean scores ranging from 3.76 to 4.17 across all domains. Meanwhile, they show a low value of authoritarian parenting and moderate authoritative parenting (mean score of 3.78), indicating that they exhibit a moderate frequency of authoritative parenting. Profile 3, with the smallest population group, holds very low expectations and places almost no emphasis on the school-readiness competence of their child, with mean scores less than 1.50 across the four domains. Their parenting style scores are low for authoritarian but also the lowest, relatively, for authoritative parenting (mean score of 3.44), suggesting the lowest frequency of authoritative parenting practice among the three profiles.

### Factors related to the latent profiles of parents’ school-readiness beliefs and parenting style

A three-step approach (Vermunt, 2010; Asparouhov and Muthén, 2014) was used in the preset study to conduct a latent profile analysis with covariates. The first step identified the latent profiles without covariates to examine the effects of covariates on profile membership, including age of child, age of respondent, birth order of child, SES and gender of the child, the second step of analysis was to derive the error terms for individuals’ assignment to their most likely latent profile. And the third step was to treat the latent profile membership as an indicator variable and examine the effects of covariates on it. The relationship between profile membership and family SES, gender, age and birth order of child is presented in Table 4.

**Table 4:** Effects of covariates on profile membership.

Profile	Covariate	Coefficient	SE	*p*-value	Odds ratio	95% confidence intervals	
	SES	−0.011	0.083	0.894	0.989	0.840	1.164
Profile 3 vs. Profile 1	Male	0.023	0.367	0.949	1.024	0.499	2.102
	Age of child	−0.022	0.028	0.423	0.978	0.927	1.033
	Only child	0.170	0.435	0.696	1.185	0.505	2.782
	First but not only child	−0.404	0.569	0.478	0.668	0.219	2.037
	Age of respondent	−0.018	0.039	0.641	0.982	0.910	1.059
	SES	−0.092	0.031	0.003*	0.912	0.858	0.970
Profile 2 vs. Profile 1	Male	0.008	0.140	0.955	1.008	0.725	1.395
	Age of child	0.009	0.011	0.406	1.009	0.987	1.032
	Only child	0.005	0.167	0.975	1.005	0.725	1.395
	First but not only child	−0.300	0.203	0.139	0.740	0.497	1.103
	Age of respondent	0.024	0.012	0.056	1.024	0.999	1.049

Reference group = Supportive parenting with a very strong emphasis on school readiness profile (Profile 1). A Bonferroni (1936) correction was applied to interpret the significance of covariates. Profiles 2 and 3 are compared to Profile 1 (the reference), leading to 2 comparisons. Thus we use an alpha of 0.025 (0.05/2 = 0.025) when determining the significance of covariates. *Denotes a significance level < 0.025.

As Table 4 shows, Profile 1 (*Supportive parenting with a very strong emphasis on school readiness*) is treated as the reference group. The odds ratios indicate the probability of the change of covariates that would be associated with the membership of a specific profile, in comparison with the reference group. The effects of covariates included in the model suggest the relative probability of being a member of Profiles 3 and 2, compared with Profile 1. As two comparisons were conducted, we used the Bonferroni correction to interpret the significance of covariates (Bonferroni, 1936), with an alpha of 0.025 (0.05/2 = 0.025). As Table 4 reveals, parents with lower SES are more likely to be in Profile 2 (*Partially supportive parenting with a reflection of school readiness*) than Profile 1 (*Supportive parenting with a very strong emphasis on school readiness*). A decrease of one unit of SES is associated with 0.09 times of increase of the likelihood of being in Profile 2, in comparison with Profile 1. However, none of the demographic characteristics are associated with profile membership.

### Parents’ attitudes regarding roles in school readiness among latent profiles

We compared the means of parents’ attitudes regarding roles in school readiness within the three latent profiles. As Table 5 shows, overall, parents with supportive parenting and a very strong emphasis on school readiness see both their own role and the school’s role in school readiness as more important than the other two subgroups. Parents with weakly supportive parenting and no emphasis on school readiness score the lowest on both their own responsibility and the school’s responsibility for their child’s school readiness. Wald tests show significant differences among the latent profiles with regard to parents’ attitudes toward roles in school readiness.

**Table 5:** Means of parents’ attitudes regarding roles in school readiness among the latent profiles.

Parents’ attitudes regarding roles in school readiness	Profile 1 Supportive parenting with a very strong emphasis on school readiness	Profile 2 Partially supportive parenting with a reflection of school readiness	Profile 3 Weakly supportive parenting with no emphasis on school readiness	Significant differences
Family role	4.66 (0.02)	4.23 (0.03)	3.75 (0.26)	1 > 3,1 > 2
School role	3.3 (0.04)	3.09 (0.06)	2.97 (0.22)	1 > 2
Item 1: Preparing my child for school is important to me and my family	4.75 (0.02)	4.36 (0.04)	3.77 (0.27)	1 > 2,1 > 3
Item 2: Preparing my child for school will help my child succeed later in school	4.59 (0.02)	4.07 (0.05)	3.74 (0.27)	1 > 3,1 > 2
Item 3: Preparing my child for school is my responsibility as a parent	4.65 (0.02)	4.26 (0.04)	3.74 (0.27)	1 > 3,1 > 2
Item 4: Preparing my child for school is the responsibility of kindergarten teachers	3.45 (0.04)	3.23 (0.06)	3.16 (0.23)	1 > 2
Item 5: Preparing my child for school is the responsibility of the primary school	3.15 (0.05)	2.95 (0.06)	2.77 (0.26)	1 > 2

A Bonferroni (1936) correction was applied to interpret the results of Wald tests. We compared the mean scores on family role and school role three times (1 v. 2, 1 v. 3, 2 v. 3). Thus, we use an alpha of 0.025 (0.05/3 = 0.017) when determining significant differences of means. Statistically significant differences among the profiles (*p* < 0.017).

## Discussion

### Patterns of parents’ school-readiness beliefs and parenting style

In the present study, three subgroups of Chinese parents were identified with regard to their school-readiness beliefs and parenting style: *Supportive parenting with a very strong emphasis on school readiness*, *Partially supportive parenting with a reflection of school readiness*, and *Weakly supportive parenting with no emphasis on school readiness*. The most prevalent subgroup was *Supportive parenting with a very strong emphasis on school readiness*, while the smallest subgroup was *Weakly supportive parenting with no emphasis on school readiness*. As both school-readiness beliefs and parenting style influence a child’s school readiness, characterizing parents based on the combination of these two factors could help to identify nuanced risks for a child’s school readiness in family contexts and support effective parental intervention programs. To our knowledge, few previous studies have explored latent profiles based on parents’ school-readiness beliefs and parenting style, thus the findings of this study are a meaningful contribution to the existing literature. Moreover, beyond the association between level of parental school-readiness beliefs and the authoritative parenting revealed in present study with a person-centered approach, our study adds to the literature by delineating in detail the major patterns of Chinese parents’ school-readiness beliefs, which is inadequately explored in prior studies. Most previous studies concerning patterns of school-readiness beliefs of parents were conducted in Western countries with different cultures from China. Our study enriches the existing literature in terms of the cultural variation of parents’ school-readiness beliefs. The patterns of Chinese parents’ school readiness beliefs and potential underlying association with Chinese contexts are discussed in this section as well.

The qualitatively different configurations of variables about school-readiness beliefs and parenting style across the three subgroups identified in the present study are expressed in two ways, as level differences and as shape differences (Spurk et al., 2020). Firstly, the most dramatic configurational differences across the three profiles are the level differences of mean values of school-readiness expectation and authoritative parenting frequency. These level differences are somewhat consistent with prior findings in a recent US study, which revealed roughly two groups of US parents regarding their school-readiness beliefs: one with a high expectation or emphasis on a child’s competence and the other with a very high expectation or emphasis (Slicker et al., 2021). However, our results are somewhat inconsistent with the three typologies of US parents’ school-readiness beliefs identified via cluster analysis by Kim et al. (2005): “Typical,” “High standards” and “Low academic emphasis,” of which the “Typical” subgroup was the largest. However, in our study, parents with a very strong emphasis on children’s school-readiness skills constitute the largest subgroup. The differences between our results and the findings of the aforementioned two studies could be derived from differences both in the parents’ country of origin and in the structure of measurement. Measurement in the present study covers four domains of school-readiness beliefs, thus allowing for the delineation of a more detailed pattern, compared to the measurement of only 6 to 7 items covering 2 to 3 domains in the two other studies. Besides, a variable-centered study showed that Chinese parents held school-readiness beliefs with a high expectation of children’s competence (Sy and Schulenberg, 2005), which is consistent with our findings that the most prevalent group very strongly emphasize their child’s school-readiness skills. It is worth noting that in our study we identified one more subgroup of parents with a very low emphasis on children’s school-readiness competence and the lowest level of authoritative parenting, which was seldom reported in prior studies. This subgroup was not identified in previous findings on Chinese parents’ school-readiness beliefs and parenting style.

Despite the level differences across the three subgroups, some nuanced shape differences, though not dramatic, are also found across three profiles, which partially support our hypotheses. Parents with *supportive parenting with a very strong emphasis on school readiness* view academic skills as the least important, relatively, and attach the greatest importance to social-emotional competence. A similar pattern is displayed by parents with *partially supportive parenting with a reflection of school readiness*, who place the least emphasis on academic skills and the greatest emphasis on approaches to learning and self-regulatory skills. The group with *Weakly supportive parenting with no emphasis on school readiness* evinces a different pattern, with the highest importance attached to academic skills and the lowest importance attached to social-emotional competence. Such results are somewhat consistent with the mixed findings in previous variable-centered research exploring Chinese parents’ school-readiness beliefs, which showed that Chinese parents stress motivation and persistence (Luo et al., 2013), or stress approaches to learning more than academic skills (Zhang et al., 2008). In a recent person-centered study, Sawyer et al. (2022) found that Chinese immigrant parents are more likely to attach great importance to learning-related skills, including approaches to learning and self-regulatory skills, rather than academic skills. Such findings support the patterns we identified in the two most prevalent subgroups in the present study. Such patterns could be associated with the Chinese cultural tradition, especially influenced by Confucian beliefs, which highlight the importance of knowledge acquisition, self-discipline, and the conformation to social norms (Luo et al., 2013; Sawyer et al., 2022). Apart from the potential impact of country-specific cultural beliefs, the emphasis on social-emotional, approaches to learning and self-regulatory competence in lieu of academic skills could be a result of the Chinese Ministry of Education’s initiative to raise awareness and dispel the myth of school readiness for parents over the past decade. Contrasted with the emphasis on academic achievement and adulted-centered learning rooted in Chinese culture, profoundly impacted by Confucianism, the policies of Chinese preschool education underscore a divergent approach. The most important national official policy guidelines strongly endorse the play-based learning for its value on promoting children’s development in five key domains, physical health, language, social, science and Art (Bautista et al., 2021). For example, Early learning and developmental guidelines for children aged 3–6 years (Ministry of Education of China, 2012) outline the learning expectations for preschool children, especially prioritizing the development attitudes and learning interests over academic knowledge gains. The “Outlines for kindergarten education (trial outlines)” emphasizes that play shall be the major activities to support children’s development (Ministry of Education of China, 2001). The aforementioned policy documents reflect an ongoing effort to mitigate the long-standing emphasis on academic skills in Chinese kindergartens. And such efforts also extended to influencing parents’ beliefs. For instance, the annual Preschool Education Promotion Month, a national event, aims to enhance parents’ knowledge about the importance of social-emotional competence, approaches to learning and self-regulatory skills for children’s school entry, as well as their awareness of the potential negative effect of over-emphasizing academic skills before school entry in the long run (Ministry of Education of China, 2016, 2019). The major pattern of school readiness beliefs revealed in present study seems to echo the school readiness skills advocated by these official documents and initiatives. However, whether such a pattern of school-readiness beliefs is derived partly from the policy effect still needs to be tested and is beyond the scope of the present study.

In overall, it’s worth noting that our findings of latent profile analysis have mainly shown the level differences rather than dramatic shape differences across three profiles. Thus, our hypotheses are only partially supported. Specifically, based on the qualitative findings by Tobin and Arzubiaga (2013), we hypothesized that one subgroup of parents would exhibit academic-oriented school-readiness beliefs and such a pattern could be related to lower SES. It is shown that parents in Profile 3, displaying *Weakly supportive parenting with no emphasis on school readiness*, value the academic skills of children slightly more than other domains, aligning with the findings of one similar study in the US (Slicker et al., 2021). However, such a pattern is not dramatic. These findings are, to some extent, surprising and counterintuitive. Compared to the study by Slicker et al. (2021) in the US, the number of parents in our study identified in Profile 3 might be too low (3%) to depict significant difference among different domains of school-readiness beliefs. Nevertheless, this pattern is consistent with previous literature, showing that there is a small proportion of Chinese parents who value academic competence more than other domains. Given that our hypothesis for the shape differences regarding parents’ school-readiness beliefs is inadequately supported in the current study, further research might be needed, with oversampling of low SES parents, to confirm the shape differences of parents’ school-readiness beliefs in China, and especially the more academic-oriented pattern with a much higher rate.

What we shall highlight is that, despite the consistency with prior findings, we found that a small proportion of parents feature more academic-oriented tendencies with a de-emphasis on social-emotional competences and the lowest level of authoritative parenting, which is different from prior findings. Disparities between previous research findings and the results of the present study could be derived from differences in two areas. Firstly, previous studies investigated Chinese immigrant parents living in the US (Sy and Schulenberg, 2005; Luo et al., 2013; Sawyer et al., 2022) or parents in other Chinese provinces (Zhang et al., 2008) with relatively small sample sizes. The socioeconomic status of parents in the previous studies was relatively high. However, the present study drew on a relatively large sample of parents characterized by a wider spectrum of socioeconomic status, thus yielding more diverse results. Secondly, a person-centered approach focuses on identifying groups of people in a population based on certain variables. However, a variable-centered approach aims to explore the distribution of certain variables. Given this study’s finding that parents who attach very low importance to a child’s competence for school readiness and are more academic-oriented and display the lowest level of authoritative parenting constitute a small proportion in the population, such an effect could be easily overlooked or averaged out in the studies with a variable-centered approach. Though this subgroup only consists of a small proportion of parents, it should not be ignored. In that sense, the findings of the present study give a more complete depiction of the characteristics of parents’ school-readiness beliefs and parenting style.

Parents’ school-readiness beliefs are associated with a child’s competences for school readiness, as revealed by a number of studies (Barbarin et al., 2008; Puccioni, 2015, 2018). Meanwhile, the positive effect of authoritative parenting and negative effect of authoritarian parenting on a child’s school readiness is revealed by previous research (Kessler, 2002; Roopnarine et al., 2006; Gao et al., 2015; Xia et al., 2020). One person-centered study shows that profiles with a higher expectation and higher frequency of parental engagement in home learning activities are associated with better school readiness, what’s more, very high expectations of parents could outweigh the importance of home learning engagement and even compensate for moderate home learning activities (Slicker et al., 2021). Moreover, robust evidence in a recent study shows that parents’ beliefs concerning with that their investments could impact child development are malleable through home visiting programs and increased beliefs are correlated with better school readiness of children (List et al., 2021). A higher level of parents’ school readiness beliefs is associated with more home literacy involvement (Brinkley et al., 2023) and more school-based involvement in the first grade (Boyle and Benner, 2020). Although our study did not examine how the distal outcomes such as children’s school readiness are associated with profile memberships, on the basis of prior findings, it could be inferred that the combined positive effect of parents’ school-readiness beliefs and authoritative parenting would lead to more favorable outcomes for children of parents in the subgroup of *Supportive parenting with a very strong emphasis on school readiness* compared to the other two groups. Conversely, the third subgroup, *Weakly supportive parenting with no emphasis on school readiness*, could be associated with more disadvantages in a child’s school readiness. However, such an assumption needs further evidence, especially in the Chinese context.

### SES and patterns of parents’ school-readiness beliefs and parenting style

SES is found to be associated with school-readiness belief profile memberships in current study, which supports our hypothesis. Parents with higher SES are more likely to display the characteristics of *Supportive parenting with a very strong emphasis on school readiness*, in comparison with the subgroup of parents with *Partially supportive parenting with a reflection of school readiness*. However, none of these factors are associated with membership in the subgroup of *Weakly supportive parenting with no emphasis on school readiness*. To our knowledge, previous research did not directly address the association between SES, parents’ school-readiness beliefs and parenting style by using a quantitative approach based on a large sample size.

Previous studies yielded mixed results in terms of the association between SES and parents’ school-readiness beliefs (Piotrkowski et al., 2000; Kim et al., 2005; Barbarin et al., 2008; Puccioni, 2015; Slicker et al., 2021; Sawyer et al., 2022). Among these studies, the results of the present study confirm the research findings of two studies (Kim et al., 2005; Slicker et al., 2021). Kim et al. (2005) adopted a person-centered approach based on a large sample size and found that US parents holding “High standards” beliefs about children’s school-readiness competence reported having a higher income and education level in comparison with parents in the “Typical” school-readiness belief group (Kim et al., 2005). Slicker et al. (2021) revealed with their latent profile analysis based on parents’ school-readiness beliefs and home learning activities, drawing on a large sample, that parents with a higher SES level are more likely to display a higher expectation of the importance of children’s school-readiness competence. Other studies have multiple limitations in different ways, especially a sample size that was small (Barbarin et al., 2008; Sawyer et al., 2022) or restricted to a population with a certain SES level (Piotrkowski et al., 2000; Barbarin et al., 2008), etc. Thus, given that both the present study and the above two studies adopted a person-centered approach based on large sample size, more support is lent for the positive association between SES and parents’ higher expectation of the importance of children’s school readiness. We did not find the association between SES and the profile membership of *Weakly supportive parenting with no emphasis on school readiness* as hypothesized, which might be derived from our sample restrictions. However, further studies need to explore this association in other populations of parents.

The results of the present study reveal that the parent profile featuring higher authoritative parenting is related to higher SES, which confirms prior variable-centered studies’ findings that higher SES was related to more authoritative parenting and less authoritarian parenting, whereas lower SES was found to be related to less authoritative and more authoritarian parenting (Bradley and Corwyn, 2002; Luo et al., 2019; Xia et al., 2020). Additionally, some nuanced characteristics of parenting style profiles were found in the present study, namely, that authoritarian parenting is low for all three groups of parents, suggesting Chinese parents tend to use less harsh and punitive parenting practices nowadays. This trend is also reported in extant literature. Although authoritarian parenting was previously reported to be a more salient feature for Chinese parents compared to their Western counterparts (Chen et al., 1997), more recent studies show that Chinese parents increasingly display more features of authoritative parenting due to the influence of contemporary child-rearing ideology (Li and Xie, 2017).

The association between higher SES and *Supportive parenting with a very strong emphasis on school readiness* shown in this study contributes to the existing literature by lending evidence to the association between SES, parents’ school-readiness beliefs and parenting style. Our finding is partially consistent with the qualitative findings by Lareau (2002, 2003). In her qualitative work, Lareau pointed out that different child-rearing “cultural logics” are held by middle-class and working-class (or poor) families. “Concerted cultivation,” featuring high dedication to supporting children’s cognitive and social development as well as reasoning and negotiation with children, constitutes the main characteristic of middle- and upper-class parents’ parenting strategies (Lareau, 2002, 2003). These attributes reflect a combination of parents’ high emphasis on the importance of child’s competences and the adoption of parenting practices similar to authoritative parenting. Our study results provide quantitative evidence for the association between higher SES and the characteristics of concerted cultivation, a combination of higher emphasis on their child’s development and authoritative parenting. Meanwhile, the high importance attached by this group of parents to their own roles in children’s school readiness echoes such findings as well. Thus, though two decades have passed since Lareau’s work was published, despite comparing the US and China, similar patterns of parenting beliefs and practices could be linked to SES.

## Implications

The results of the present study could lend support to parental intervention programs, especially for the minority population of Chinese parents who have very low expectations with almost no emphasis on their child’s school-readiness competence and engage in authoritative parenting least frequently. As revealed in our results, the risks related to a child’s school readiness could be doubled for the above subgroup, and so this pattern of school-readiness beliefs and parenting style should be of the greatest concern for parental intervention programs. Such targeted intervention programs conducted in the US, particularly for disadvantaged families, show positive effects on a child’s school readiness by enhancing supportive parenting, parental engagement and building up parent–teacher collaboration. However, these intervention programs evince a potential association between lower SES and decreased parental attendance in the program, while the necessity of matching the needs of the diverse backgrounds of targeted families is stressed (Sheridan et al., 2010; Marti et al., 2018). Thus, it is necessary to offer diverse support in intervention programs for targeted families, especially those with lower-SES backgrounds. Besides, three subgroups of parents with different school-readiness beliefs and parenting style suggest that a person-centered transition towards support and collaboration between the family, the preschool institution and the primary school is necessary. For instance, given that *Supportive parenting with a strong emphasis on school readiness* constitutes the largest subgroup in the present sample and that the transition to school should tackle the barriers to mutual understanding and collaboration between parents, preschools and primary schools to facilitate continuity for children, such a major pattern of parents’ school-readiness beliefs and parenting style could help schools to better understand parents and build up mutual understanding with parents from a cultural perspective.

## Limitations and future directions

This study’s strengths lie in several areas. Firstly, the large sample size, greater than the minimum recommended size of 500 (Nylund et al., 2007), allowed us to identify subgroups via LPA with sufficient accuracy. Secondly, the sample covers a wide range of SES to allow us to explore the association between SES and patterns of school-readiness beliefs and parenting style. Lastly, with a powerful person-centered approach, this study simultaneously examines profiles based on a combination of contributive factors and risks related to children’s school readiness, as well as the covariates associated with the given profile membership, which was seldom addressed before.

However, this research is not without limitations. Results should be interpreted and generalized with caution. Though we drew on a large sample of parents in Chongqing, such a sample is far from representative of all Chinese parents as China has a very large population covering vast geographic and socio-cultural diversities; the three patterns identified in our study might not be applicable to parents in other areas. Consequently, future research shall examine whether these patterns are to be found in other samples. Additionally, some methodological limitations in the present study are also worth considering. The self-reported school-readiness beliefs and parenting style could be biased due to their perceived social desirability, and their reliability could be compromised by certain response sets from parents. Further evidence from observational data is needed. For the potentially most disadvantaged subgroup identified in this study, which displays very low expectations and the lowest level of authoritative parenting, we failed to identify factors associated with membership in the profile. Further studies should explore with in-depth interviews or take other covariates into consideration, such as a child’s development delay, parents’ personal educational experience, etc. Finally, distal outcomes, such as how well children adapt and how parents are committed to helping children during transition to school, associated with the three patterns of school-readiness beliefs and parenting style should be explored to lend evidence to the predictive validity of such patterns.

## Data availability statement

The raw data supporting the conclusions of this article will be made available by the authors, without undue reservation.

## Ethics statement

Ethical review and approval was not required for the study on human participants in accordance with the local legislation and institutional requirements. Written informed consent was not required to participate in this study in accordance with the local legislation and institutional requirements.

## Author contributions

SC: Writing – original draft, Conceptualization, Data curation, Methodology, Writing – review & editing. DG: Writing – review & editing, Conceptualization, Funding acquisition, Methodology, Supervision, Writing – original draft.
