# Supplementary material for: Chinese parents’ school-readiness beliefs and parenting styles: patterns and associated factors
Source: Front Psychol. 2024 Jan 9;14:1279175. doi: 10.3389/fpsyg.2023.1279175 (PMC10803651; doi:10.3389/fpsyg.2023.1279175)
Supplement: Supplementary file 1 [file Table_1.DOCX]

**School readiness belief scale**

For the following items, on a scale of 1 to 5, where 1 = not important and 5 = very important

, choose a number to indicate “how important do you think it is for a child starting school.”

| **Dimensions** | Items |
| --- | --- |
| ***Academic competence*** |  |
|  | Writes words other than his/her name. 会写自己名字以外的字。 |
|  | Knows most letters of alphabets/many characters. 认识大部分拼音字母/许多汉字。 |
|  | Counts by himself/herself. 会自己数数。 |
| ***Approaches to learning*** |  |
|  | Is self-confident. 对自己有自信。 |
|  | Has patience. 有耐心。 |
|  | Is curious, asks lots of questions about how and why. 好奇，问很多为什么，怎么样的问题。 |
| ***Self-regulatory competence*** |  |
|  | Is not disruptive of the class. 不扰乱课堂。 |
|  | Sits still and pays attention to teacher. 能坐好并注意听讲。 |
|  | Completes tasks on time. 按时完成任务。 |
|  | Follows directions. 听从指令。 |
| ***Social emotional competence*** |  |
|  | Takes turns and shares. 会轮流和分享。 |
|  | Communicates needs/wants verbally. 能口头表达自己的需要。 |
|  | Has good problem-solving skills with peer relations. 会解决同伴交往中遇到的问题。 |
|  | Shows respect for others. 尊重他人。 |

**Parents’ attitudes toward roles in school readiness scale**

Please read the following statements, express the extent to which you agree with each statement, on a scale of 1 to 5, where 1 = Disagree and 5 = very much agree.

| Dimensions | Items |
| --- | --- |
| Family role |  |
|  | Preparing my child for school is important to me and my family. 为孩子做好入小学的准备对于我和家人来说是一件重要的事情。 |
|  | Preparing my child for school will help my child succeed later in school为孩子做好入小学的准备能够帮助他/她以后学业取得成功。 |
|  | Preparing my child for school is my responsibility as a parent 为孩子做好入学准备是我作为父母的责任。 |
| School role |  |
|  | Preparing my child for school is the responsibility of kindergarten teachers为孩子做好入学准备是幼儿园的责任。 |
|  | Preparing my child for school is the responsibility of the primary school 为孩子做好入学准备是小学的责任。 |

**Authoritarian parenting subscale**

Please read the following items and think about how often you engage in the different parenting practices listed below. Rate from 1 = “Never” and 5 = “Always”, to indicate the frequency of which you do the following things.

| Dimensions | Items |
| --- | --- |
| Physical coercion |  |
|  | Yell or shout when child misbehaves.当孩子不听话的时候打他/她屁股。 |
|  | Grab child when being disobedient.当孩子不服从的時候，会抓住孩子，不让他/她乱动。 |
|  | Slap child when the child misbehaves. 当孩子行为不当的时候，用手掌拍打孩子。 |
| Verbal hostility |  |
|  | Yell or shout when child misbehaves.当孩子做错的時候，对着孩子吼叫。 |
|  | Explode in anger towards child.对孩子大发雷霆。 |
|  | Scold and criticize to make child improve.为了促使孩子进步，会责备及批评他/她。 |
|  | Scold and criticize when child’s behavior doesn’t meet our expectations.当孩子未能达到我的期望，会责骂或批评他/她。 |
| Punitive dimension |  |
|  | When child asks why (he)(she) has to conform, state: because I said so, or I am your parent and I want you to.当孩子问他/她为什么必须服从的时候，对孩子说：“因为这是我说的”，或“因为我是你的父母，我想让你这样做。” |
|  | Punish by taking privileges away from child with little if any explanations.几乎不进行任何解释，就把孩子原本享有的特权拿走，借以惩罚孩子。 |
|  | Use threats as punishment with little or no justification.在沒有充分理由的情况下，用威胁来惩罚孩子。 |
|  | Punish by putting child off somewhere alone with little if any explanations.在沒有充分理由的情况下，把孩子单独放在某个地方以示惩罚，例如不让出房门。 |

**Authoritative parenting subscale**

Please read the following items and think about how often you engage in the different parenting practices listed below. Rate from 1 = “Never” and 5 = “Always”, to indicate the frequency of which you do the following things.

| Dimensions | Items |
| --- | --- |
| Connection |  |
|  | Responsive to child’s feelings or needs.会回应孩子的感受及需要。 |
|  | Encourage child to talk about the child’s troubles.鼓励孩子说出他/她的烦恼。 |
|  | Give comfort and understanding when child is upset.当孩子伤心难过時，给予理解和安慰。 |
|  | Give praise when child is good.当孩子表现好的時候给予表扬。 |
|  | Have warm and intimate times together with child.与孩子之间有关系亲密温暖的时刻。 |
| Regulation |  |
|  | Explain to child how we feel about the child’s good and bad behavior.向孩子解释我们对他/她好的行为和坏的行为有怎样的感受。 |
|  | Emphasize the reasons for rules.强调规则背后的理由。 |
|  | Give child reasons why rules should be obeyed.向孩子解释为什么要遵守规则。 |
|  | Help child to understand the impact of behavior by encouraging child to talk about the consequences of his/her own actions.通过鼓励孩子谈论自己的行为后果，来帮助孩子理解他/她的行为所造成的影响。 |
|  | Explain the consequences of the child’s behavior.向孩子解释他/她的行为会产生的后果。 |
| Autonomy |  |
|  | Take child’s desires into account before asking the child to do something.在要求孩子做某件事前，会考虑他/她的意愿。 |
|  | Encourage child to freely express (him/herself) even when disagreeing with parents.即使跟孩子跟父母意见不同，也鼓励他/她表达自己的想法。 |
|  | Take into account child’s preferences in making plans for the family.在做家庭计划时，会考虑孩子的喜好。 |
|  | Show respect for child’s opinions by encouraging child to express them.鼓励孩子表达自己的想法以示尊重他/她的意见。 |
|  | Allow child to give input into family rules.让孩子对家规提意见。 |
