# Supplementary material for: Chinese parents’ school-readiness beliefs and parenting styles: patterns and associated factors
Source: Front Psychol. 2024 Jan 9;14:1279175. doi: 10.3389/fpsyg.2023.1279175 (PMC10803651; doi:10.3389/fpsyg.2023.1279175)
Supplement: Supplementary file 2 [file Data_Sheet_1.docx]

Supplementary Tables

Table 1 Correlation matrix of main variables

|  | 1 | 2 | 3 | 4 | 5 | 6 | 7 | 8 | 9 |
| --- | --- | --- | --- | --- | --- | --- | --- | --- | --- |
| 1.Age of respondents | 1.00 |  |  |  |  |  |  |  |  |
| 2.Age of child | 0.07* | 1.00 |  |  |  |  |  |  |  |
| 3.SES | 0.15*** | 0.2 | 1.00 |  |  |  |  |  |  |
| 4.Academic | -0.08** | -0.05 | -0.12*** | 1.00 |  |  |  |  |  |
| 5.Social-emotional | -0.04 | 0.00 | 0.06* | 0.73*** | 1.00 |  |  |  |  |
| 6.Self-regulatory | 0.00 | -0.02 | 0.02 | 0.71*** | 0.88*** | 1.00 |  |  |  |
| 7.Approaches to learning | -0.01 | 0.04 | 0.14*** | 0.61*** | 0.78*** | 0.73*** | 1.00 |  |  |
| 8.authoritarian | -0.06 | 0.00 | -0.4*** | 0.04 | 0.00 | -0.01 | -0.05 | 1.00 |  |
| 9.authoritative | -0.02 | 0.00 | 0.28*** | 0.22*** | 0.37*** | 0.30*** | 0.31*** | -0.14*** | 1.00 |

Table 2 Descriptive statistics for indicators of latent profile analysis

|  | M(SE) | Skewness | Kurtosis |
| --- | --- | --- | --- |
| Academic | 4.27(0.02) | -1.55 | 3.07 |
| Social-emotional | 4.58(0.02) | -3.2 | 12.53 |
| Self-regulatory | 4.60(0.02) | -3.13 | 11.73 |
| Approaches to learning | 4.52(0.02) | -2.84 | 9.17 |
| Authoritarian | 2.12(0.02) | 1.3 | 2.71 |
| Authoritative | 4.11(0.02) | -0.75 | 1.3 |

Note: Item averaged score is used, all the scores range from 1 to 5.
